# Efficient Charge Carrier Separation in l-Alanine Acids Derived N-TiO_2_ Nanospheres: The Role of Oxygen Vacancies in Tetrahedral Ti^4+^ Sites

**DOI:** 10.3390/nano9050698

**Published:** 2019-05-05

**Authors:** Yongjuan Chen, Xiu Luo, Yao Luo, Peiwen Xu, Jiao He, Liang Jiang, Junjie Li, Zhiying Yan, Jiaqiang Wang

**Affiliations:** School of Chemical Sciences & Technology, National Center for International Research on Photoelectric and Energy Materials, Yunnan Provincial Collaborative Innovation Center of Green Chemistry for Lignite Energy, Yunnan Province Engineering Research Center of Photocatalytic Treatment of Industrial Wastewater, The Universities’ Center for Photocatalytic Treatment of Pollutants in Yunnan Province, Yunnan University, Kunming 650091, China; chenyongjuan@ynu.edu.cn (Y.C.); xiuluo@ynu.edu.cn (X.L.); LYjdt123@163.com (Y.L.); juneya@126.com (P.X.); hejiao@ynu.edu.cn (J.H.); liangjiang_ynu@163.com (L.J.); junjieli@ynu.edu.cn (J.L.); zhyyan@ynu.edu.cn (Z.Y.)

**Keywords:** N-doped TiO_2_, tetrahedral Ti^4+^ sites, photocatalytic, l-alanine acids, time-resolved IR spectroscopy

## Abstract

N-doped TiO_2_ with oxygen vacancies exhibits many advantages for photocatalysis, such as enhanced visible light absorbency, inhibition of the photogenerated charge carrier recombination, etc. However, preparation of N-doped TiO_2_ with oxygen vacancies under mild conditions is still a challenge. Herein, N-doped TiO_2_ nanospheres with tetrahedral Ti^4+^ sites were synthesized by using dodecylamine as template and assisted by l-alanine acids. The obtained samples were characterized by X-ray powder diffraction (XRD), scanning electron microscopy (SEM), transmission electron microscopy (TEM), and UV–Vis diffuse reflectance spectra (UV–Vis DRS). It was found that the dodecylamine as a neutral surfactant controlled the structure of TiO_2_ spherical, while l-alanine acids provided a nitrogen source. The existence of tetrahedral Ti^4+^ sites in N-doped TiO_2_ was also confirmed. The N-doped TiO_2_ sample with tetrahedral Ti^4+^ sites exhibited significantly improved photocatalytic performance for degradation of methylene blue solution under UV light or visible light irradiation. A combined time-resolved infrared (IR) spectroscopy study reveals that the enhanced photocatalytic performance could be attributed to a large amount of photogenerated charge carriers and efficient charge separation. It is demonstrated that the shallow donor state produced by oxygen vacancies of tetrahedral Ti^4+^ sites can effectively promote separation of charge carriers besides capturing electrons.

## 1. Introduction

TiO_2_-based photocatalysts have been most widely investigated in the past decades. However, practical applications of TiO_2_ are still very limited because its fast recombination of photogenerated electron-hole pairs and wide band gap (3.2 eV) lead to a low quantum efficiency of photocatalytic reaction. Various strategies have been developed for improving the photocatalytic activities of TiO_2_ based photocatalysts, such as combined with a semiconductor, metal ion/nonmetal doping, deposition with noble metal on surface, dyes sensitization, and defect creation [1]. Doping of TiO_2_ with nitrogen was considered as a promising approach to improve photocatalytic activity for TiO_2_ under visible light [2]. A number of strategies can be used to prepare N-doped TiO_2_, for example, sputtering [3], ion implantation [4], chemical treatments of TiO_2_ [5], sol–gel process [6], thermal oxidation from TiN [7], etc. The sol–gel method is usually preferred because doping amount of N and the size of the sample can be easily controlled [8]. In this doping process, some specific acid or alkali additives can act as a cotemplate along with surfactants/polymer assemblies and this may play a significant role in precisely tailoring both TiO_2_ structure and morphology [9]. Kanie et al. [10] found that ammonia and primary amines are more likely to make TiO_2_ generate elliptical particles in gel–sol process. Durupthy et al. [11] also demonstrated the presence of the amino acids has an effect on the kinetics and thermodynamics during the crystalline TiO_2_ formation. These accomplishments have inspired us to use amino acids in preparing N-doped TiO_2_ with unique structure, and amino acids can also serve as good sources of nitrogen.

In process of N-doping, oxygen vacancies often occur in the lattice of titanium dioxide [12,13]. The stability of surface oxygen vacancies may lead to improvement of photocatalytic activity. Krol et al. [14] revealed that coordination geometry, from an octahedral to a tetrahedral of Ti^4+^ in TiO_2_, can be adjusted by generating a large number of stable oxygen vacancies. Gray et al. [15] also confirmed that the tetrahedral Ti^4+^ sites contribute to the increased photoactivity. Therefore, the construction of tetrahedral Ti^4+^ sites is also an effective strategy to produce stable oxygen vacancies. But treatment at high-temperature by used reducing gases (e.g., NH_3_ [16] and H_2_ [17]) is required. Thus, it is still a challenge to develop synthetic strategy of tetrahedral Ti^4+^ sites under mild conditions and exploiting their unique properties for photocatalytic applications. 

Herein, we describe a new and facile chemical process to synthesize N-doped TiO_2_ nanospheres with tetrahedral Ti^4+^ sites using dodecylamine as template and assisted by l-alanine acids. The photocatalytic performance of the obtained samples was measured by photodegradation of methylene blue (MB) under UV and visible light irradiation. The sample with tetrahedral Ti^4+^ sites in N-doped TiO_2_ exhibited significantly photocatalytic performance, whether under UV light or visible light.

## 2. Materials and Methods

### 2.1. Synthesis of Samples

All reagents were analytical grade and used without further purification. In a typical synthesis [18], 1.83 g dodecylamine (DDA, Sinopharm Chemical Reagent Co., Ltd., Shanghai, China) was dissolved in 30 mL absolute ethyl alcohol, and 3.21 g titanium tetraisopropoxide (TTIP, ≥97%, Sigma-Aldrich, St. Louis, MO, USA) was then added slowly in this solution. Subsequently, 60 mL l-alanine acids (Ala, Sinopharm Chemical Reagent Co., Ltd., Shanghai, China) solution (0.187 mol·L^−1^) was added dropwise stirring at 333 K for 24 h. The final mixture was transferred into a Teflon bottle (250 mL) and heated at 363 K for 96 h under autogenous pressure. After the autoclave was cooled to room temperature, the slurry was filtered and washed with deionized water for several times. Finally the product was calcined at 673 K for 4 h in muffle furnace. The obtained example was denoted as MTiO_2_/Ala-DDA (M represents mesoporous). A similar procedure was applied in the preparation of MTiO_2_ and MTiO_2_/Ala in the absence of l-alanine acids (Ala) and dodecylamine (DDA), respectively.

### 2.2. Characterizations

X-ray powder diffraction (XRD) experiments were carried out using a Rigaku TTRIII X-ray diffractometer (Rigaku D/max-3B, Tokyo, Japan) with Cu K_α_ radiation. The Brunauer–Emmett–Teller (BET) surface area was measured on a Micromeritics Tristar II Surface Area and Porosity Analyzer (Micromeritics, Norcross, GA, USA). A pore size distribution was obtained by Barrett–Joyner–Halenda (BJH) method using nitrogen desorption data measured at 77 K. Scanning electron microscopy (SEM) images were taken by FEIQuanta200FEG microscope (FEI, Eindhoven, The Netherlands) at an accelerating voltage of 15 kV. The transmission electron microscopy (TEM) and high-resolution transmission electron microscopy (HRTEM) images were obtained by JEM Fas-TEM-3010 electron microscope instrument (JEOL, Tokyo, Japan) at the accelerating voltage of 200 kV. UV–Vis diffuse reflectance spectra (UV–Vis DRS) were measured by UV-2401PC photometer (Shimadzu, Kyoto, Japan) using BaSO_4_ as a reflectance standard. X-ray photoelectron spectroscopy (XPS) measurements were performed using a Thermo Scientific K-Alpha XPS system (Thermo Fisher Scientific, Waltham, MA, USA) equipped with a monochromatic Al K_α_ source.

### 2.3. Photocatalytic Activity

The photocatalytic activities were measured by photodegradation of methylene blue (MB, Guangzhou Chemical Reagent Factory, Guangzhou, China). The initial concentration of MB was 10 ppm (50 mL) and the amount of the photocatalyst used was 25 mg. The dispersion was magnetically stirred in the dark for several hours to achieve the adsorption/desorption equilibrium between the dye and the photocatalyst before illumination. In UV light photocatalytic experiment, two 30 W UV lamps (Philips Lighting Co, Eindhoven, The Netherlands) were used as light source. The light intensity was 25 mW∙cm^−2^. Visible light photocatalytic activities were measured under white-light LED (5 W, PCX-50C, Beijing perfectlight technology co. LTD, Beijing, China). At given irradiation time intervals, 4 mL of the suspensions were collected and centrifuged. The degraded solutions of MB were analyzed by a UV-2401PC photometer (Shimadzu, Kyoto, Japan). 

### 2.4. Time-Resolved IR Measurement

Electron decay signals were recorded by a Nicolet 8700 FTIR spectrometer with the InSb detector (Thermo Nicolet Corp., Madison, WI, USA). The frequency was 25 MHz and the scanning range is 1850 cm^−1^ to 7400 cm^−1^. The sample was photoexcited by 355 nm laser of 10 ns pulse from third harmonic generation of a Q-switched Nd: YAG laser (Labeit, Beijing, China). The pulse energy and frequency was tuned to 4 mJ and 10 Hz, respectively.

## 3. Results

### 3.1. Characterizations

**Figure 1:**
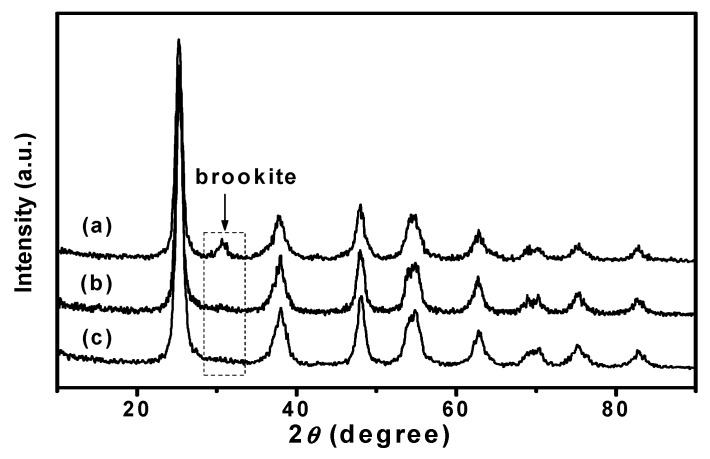
Patterns for (a) MTiO_2_/Ala, (b) MTiO_2_/DDA, and (c) MTiO_2_/Ala-DDA.

The crystalline structures of as-prepared samples were investigated by XRD measurements. The XRD patterns of MTiO_2_/Ala-DDA and MTiO_2_/DDA (Figure 1) show peaks of 2θ values at 25.3°, 37.9°, 48.0°, 54.6°, 62.7°, 75.2°, and 82.7°, which correspond to (101), (004), (200), (211), (204), (215), and (224) crystallographic planes of anatase TiO_2_ (JCPDS, No. 65-5714), respectively. No peak attributed to other phases was observed indicates the formation of pure anatase. However, MTiO_2_/Ala shows an extra peak at 2θ = 30.81°, which corresponds to the (121) crystallographic plane of brookite (JCPDS, No. 03-0380). The amount of anatase and brookite phase is 79% and 21%, respectively, calculated from the intensities of two peaks: (101) and (121) plane [19]. This indicates that l-alanine acid is the key role in the phase transition from anatase into brookite. But when l-alanine acids and dodecylamine were simultaneously added, only anatase phase was obtained. It is suggested that the existence of dodecylamine make TiO_2_ more inclined to maintain the structure of anatase.

**Figure 2:**
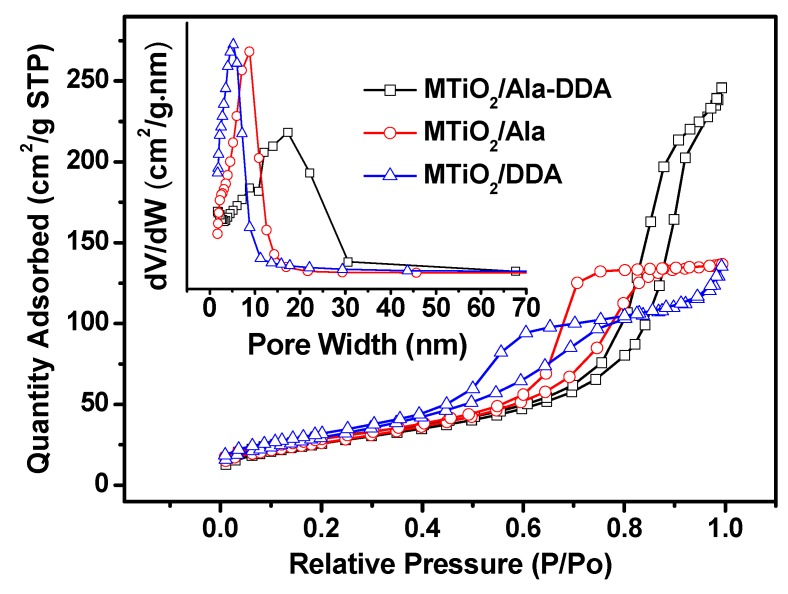
N_2_-sorption isotherms and pore size distribution curves (inset) of as-prepared samples.

Figure 2 shows the nitrogen adsorption–desorption isotherms and BJH pore size distribution plots (inset to Figure 2) for as-prepared samples. All examples exhibited type-IV isotherm, which is characteristic of mesoporous materials, but there are significant differences in the isotherm for each sample. The isotherms of MTiO_2_/Ala and MTiO_2_/DDA are intermediate between typical H1 and H2 type hysteresis loop, and the pore size distributions consists of single narrow peaks. It is implied that the materials have very uniform pore channels in the mesoporous region. The isotherm of MTiO_2_/Ala-DDA shows slightly resembles H3 type hysteresis loop, and the pore size distribution is broad. This associated with aggregates of plate like particles giving rise to slit like pores [20]. Using the BJH method and the desorption branch of the nitrogen isotherm, the average pore size of MTiO_2_/Ala-DDA, MTiO_2_/DDA and MTiO_2_/Ala is 11.8, 5.0, and 5.7 nm, respectively. The BET specific surface areas of MTiO_2_/Ala-DDA, MTiO_2_/DDA, and MTiO_2_/Ala are 97.2, 110.1, and 96.4 m^2^·g^−1^, respectively.

**Figure 3:**
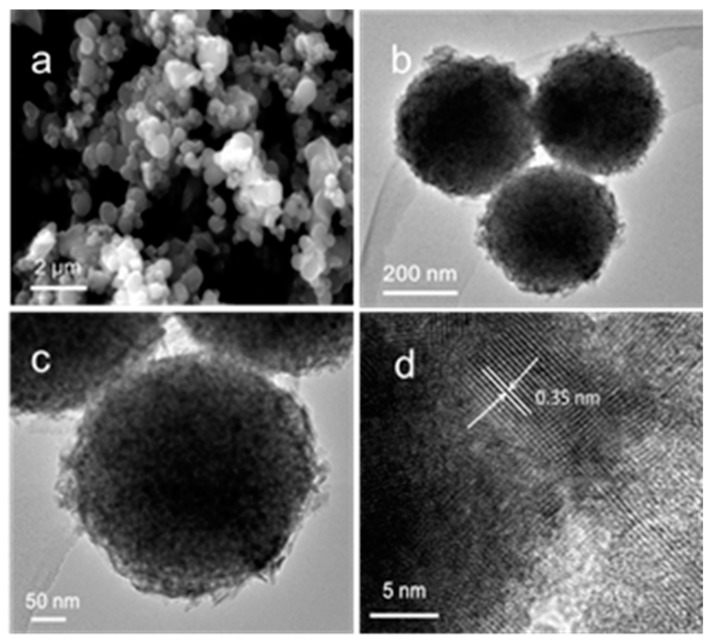
(**a**) Typical SEM, (**b**,**c**) show TEM images of MTiO_2_/Ala-DDA with different magnifications, and (**d**) shows the HTEM for MTiO_2_/Ala-DDA nanocrystallinity.

**Figure 4:**
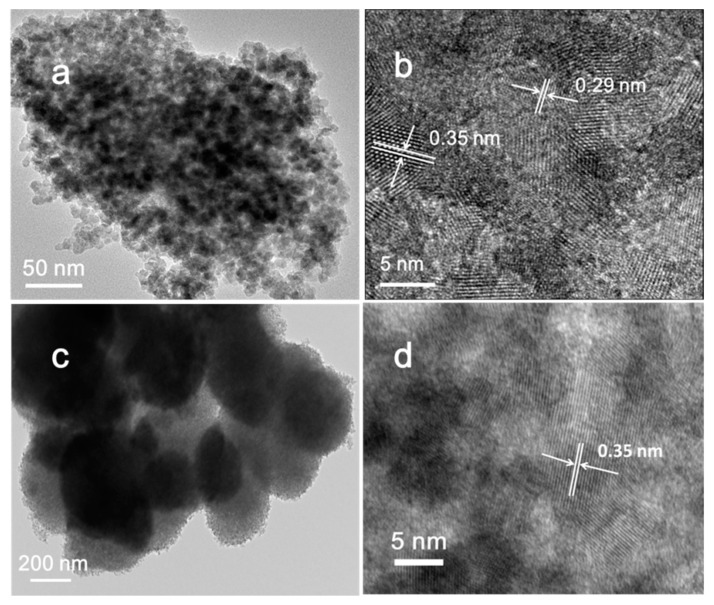
(**a**) TEM and (**b**) HTEM images of MTiO_2_/DDA. (**c**) TEM and (**d**) HTEM images of MTiO_2_/Ala.

**Scheme 1:**
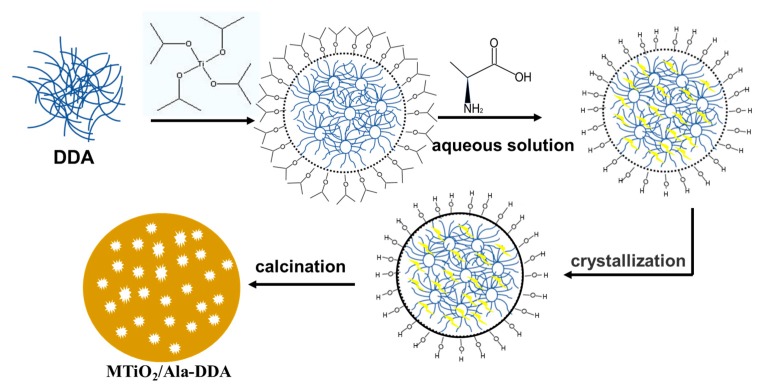
Schematic illustration for the formation of MTiO_2_/Ala-DDA.

The morphology and crystal structure of the samples were performed by SEM and TEM. Figure 3a shows the SEM image of MTiO_2_/Ala-DDA; a number of TiO_2_ spheres with diameters in the range of 300 to 500 nm were observed. As shown in Figure S1a,b, the normal distribution of MTiO_2_/Ala-DDA from 100 nm to 700 nm in diameter is consistent to the results from SEM images. Meanwhile, the irregular distribution above 700 nm should be attributed to different aggregation level of the particles. Figure 3b,c shows the TEM images of MTiO_2_/Ala-DDA with different magnifications. The TiO_2_ spheres consisted of a large number of nanoparticles. In Figure 3d, the 0.35 nm interlayer spacings of MTiO_2_/Ala-DDA corresponding to the (101) plane of anatase TiO_2_ can be obviously observed, revealing a well-defined crystal structure. Figure 4a shows TEM image of the MTiO_2_/Ala nanocrystals. The average diameter of the nanocrystals was about 10 nm. The clear lattice fringes with lattice space of 0.35 nm and 0.29 nm presented by the high-resolution transmission electron microscopy (HRTEM) image (Figure 4b) of MTiO_2_/Ala are assigned to (101) plane of anatase TiO_2_ and (121) planes of brookite, respectively. These results agree well with XRD date. As shown in Figure 4c, the morphology of MTiO_2_/DDA nanocomposite is also a spherical structure, indicates that DDA micelles tend to form a spherical shape [21]. In fact, DDA has been used as a neutral amine surfactant to control the growth of the nanocrystals because of its long nonpolar carbon chain [22]. The schematic illustration for the formation of MTiO_2_/Ala-DDA is shown in Scheme 1.

**Figure 5:**
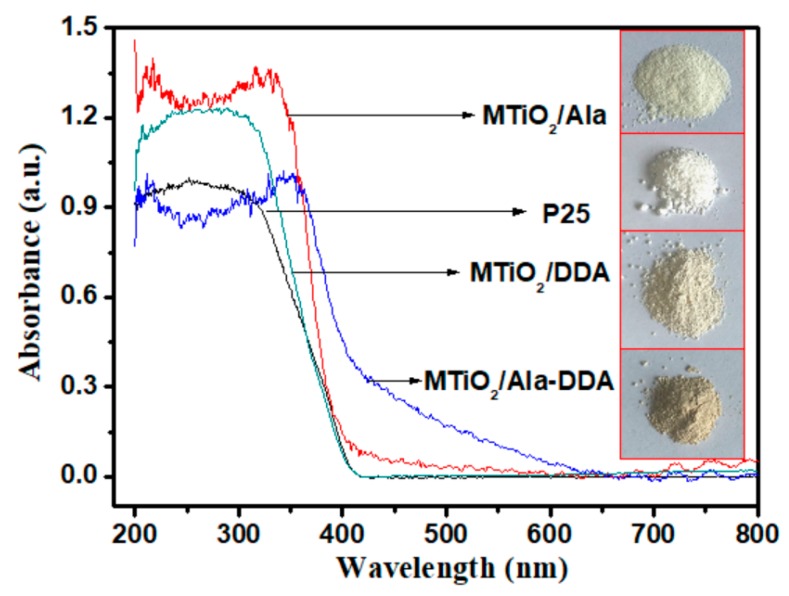
UV−Vis DRS spectra of P25, MTiO_2_/Ala-DDA, MTiO_2_/DDA, and MTiO_2_/Ala (inset shows digital camera photographs of samples).

As shown in Figure 5, the UV–Vis DRS spectra of P25 and MTiO_2_/DDA exhibit the typical absorption of TiO_2_. The absorption edge for P25 and MTiO_2_/DDA both can be determined to be around 400 nm, corresponding to band gap of 3.1 eV. However, MTiO_2_/Ala-DDA and MTiO_2_/Ala exhibit enhanced absorption in visible light region, in accordance with the colour of beige and pale yellow, respectively. Moreover, the absorption intensity of MTiO_2_/Ala-DDA in the visible light region is much stronger than that of MTiO_2_/Ala. These results indicate that MTiO_2_/Ala-DDA may be doped with other elements or has special structure.

**Figure 6:**
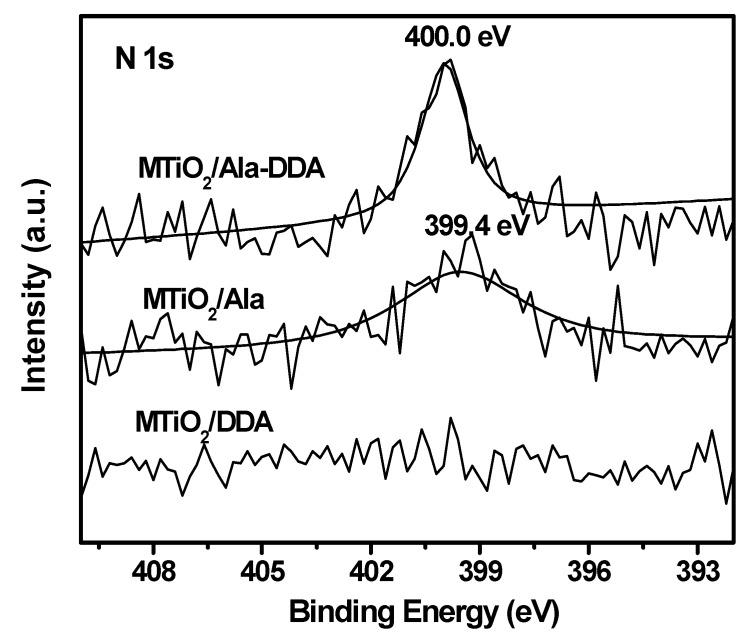
X-ray photoelectron spectroscopy (XPS) N 1s spectra of MTiO_2_/Ala-DDA, MTiO_2_/Ala, and MTiO_2_/DDA.

The surface chemical composition and chemical states of the as-prepared samples were analyzed by XPS. As shown in Figure 6, N 1s core level peak at ~400 eV was observed for MTiO_2_/Ala-DDA and MTiO_2_/Ala, whereas no N 1s signals were detected on the surface of MTiO_2_/DDA. The N 1s core level of N-doped TiO_2_ at ~400 eV should be attributed to N–O bonding (i.e., Ti–O–N) [23]. Although there are some disputes for N 1s peak alone at 399–400 eV and assigned it as N–O bonding [2]. The molecular N_2_ is not chemisorbed on metal oxides like TiO_2_ at room temperature. So, it is suggested that nitrogen was successfully doped in TiO_2_ lattice for MTiO_2_/Ala-DDA and MTiO_2_/Ala, consistent with the results of UV-Vis diffuse reflectance spectroscopy.

**Figure 7:**
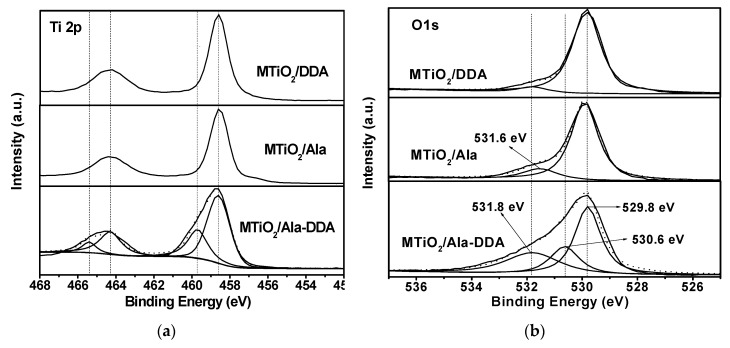
XPS (**a**) Ti 2p spectra and (**b**) O 1s spectra of MTiO_2_/Ala-DDA, MTiO_2_/DDA, and MTiO_2_/Ala.

Figure 7a shows that all samples had octahedrally coordinated Ti species with a Ti 2p3/2 peak, which was situated at lower binding energy (458.6 eV). It is interesting that MTiO_2_/Ala-DDA exhibited another contribution at higher binding energy (~459.7 eV), which is the fingerprint of tetrahedrally coordinated Ti (IV) [24,25]. The high-energy shift of Ti 2p3/2 indicated change in coordination from octahedral to tetrahedral. The tetrahedral Ti^4+^ species was confirmed to be an intermediate in the phase transformation from anatase to rutile [15], which is usually prepared in zeolite cavities or dispersed onto silica substrate [26,27]. The results of XRD shows that in the presence of only l-alanine acids lead to the formation of anatase and brookite structure, whereas in the presence of both l-alanine acids and dodecylamine, only the anatase phase were obtained. Therefore, tetrahedral Ti^4+^ species with MTiO_2_/Ala-DDA may be generated in the process of phase transformation from brookite to anatase. However, this is only a hypothesis and has not yet been confirmed. The formation of tetrahedrally coordinated Ti^4+^ species may also be due to presence of oxygen vacancies in TiO_2_ [13]. The Ti^4+^ species brought the ligand-to-metal charge-transfer (LMCT) during the photocatalytic process and generated highly active photoexcited charge-carriers, which due to the transition from [Ti^4+^—O^2−^] to [Ti^3+^—O^−^]* [28,29].

The XPS spectra of the as-prepared samples in the O1s region all show two peaks at approximately 529.8 and 531.8 eV, which can be assigned to the lattice oxygen in Ti–O bond and OH groups on surfaces of the samples, respectively (Figure 7b) [16,30]. For the MTiO_2_/Ala-DDA TiO_2_, there is another peak at 530.6 eV perhaps belonging to O_2_ molecules adsorb as O_2_^−^ on the surface of TiO_2_ when excess negative charge associated with oxygen vacancies [31,32]. This also further confirmed the formation of tetrahedrally coordinated Ti^4+^. The O_2_ adsorbed on the surface of TiO_2_ can produce superoxide radical groups by capturing photoinduced electrons as well as the free electrons located on oxygen vacancy states [33]. Thus, the present of TiO_4_ clusters by creating oxygen vacancies may lead to increasing in activities for photocatalytic reactions [14].

### 3.2. Photocatalytic Activity

**Figure 8:**
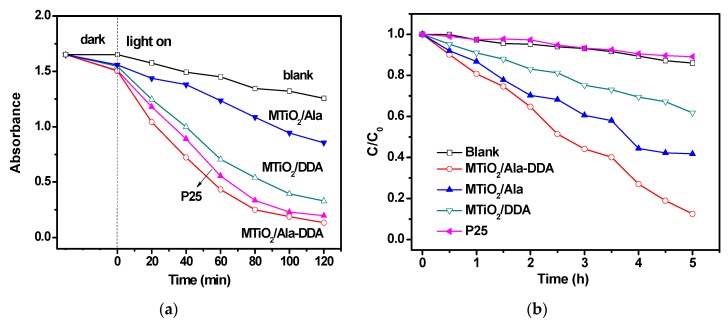
A comparison of the photoactivities of MTiO_2_/Ala-DDA, MTiO_2_/DDA, MTiO_2_/Ala, and blank experiment (without catalyst) under (**a**) UV light and (**b**) visible light.

The photocatalytic activities are measured by degradation of methylene blue (MB). After reaching adsorption equilibrium, the variations for maximum absorbance of MB are summarized in Figure 8a. It can be found that all samples show a little adsorption capacity for MB under dark. The adsorption yield of MB over MTiO_2_/Ala-DDA, MTiO_2_/DDA, MTiO_2_/Ala, and P25 is 8.9%, 6.5%, 5.6%, and 8.7%, respectively. Approximately 23% of MB was photo-decomposed in the absence of a catalyst after 120 min, indicating MB is unstable under UV irradiation. However, all the catalysts exhibited specific photocatalytic activities for MB degradation under UV light. Considering the photo-decomposition of MB itself, photodegradation yield = [(C_e_ − C_a_ − C_b_)/C_e_]*100% [34]. C_e_ is the concentration of MB when reaching adsorption equilibrium, C_a_ is the concentration after photodegradation under UV light, and C_b_ is the decrease concentration because of the direct photolysis. The photodegradation yield of MB over MTiO_2_/Ala-DDA, P25, MTiO_2_/DDA, and MTiO_2_/Ala is approximately 64.9%, 60.7%, 53.1%, and 19.8%, respectively.

The photocatalytic activities of the as-prepared catalysts were also evaluated under visible lights irradiation as shown in Figure 8b. Obviously, MB is much more stable under visible light. Only about 14% of MB was decomposed within 5 h without any catalyst. As expected, no photocatalytic activity was observed for P25 under visible light, and MTiO_2_/DDA also showed a low photocatalytic activity. However, TiO_2_/Ala-DDA and MTiO_2_/Ala exhibited significant photodegradation performances under visible light. Considering the self-sensitization of MB, colorless phenol was also selected as the simulated pollutant for evaluation of photocatalytic activities over the synthesized photocatalysts. Although the photocatalytic degradation yield of phenol over all the samples is not impressive, MTiO_2_/Ala-DDA still exhibits the highest activity for photocatalytic degradation of phenol (Figure S2). It is suggested the N-doping level had influence on the visible light photocatalytic activities of the TiO_2_ samples. Moreover, the formation of tetrahedrally coordinated Ti^4+^ in TiO_2_/Ala-DDA results in enhanced absorption in visible light region.

### 3.3. Decay Kineticas of Photogenerated Electrons

**Figure 9:**
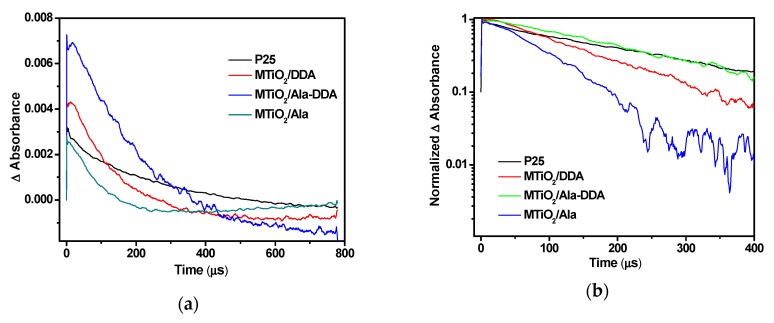
(**a**) Temporal profiles in atmosphere of transient IR absorption and (**b**) normalized decay curves at 1910 cm^−1^ of the four catalysts.

Time-resolved IR spectroscopy was demonstrated as a powerful method to accurately trace the decay of photogenerated electrons result in recombination or carrier reactions. Infrared absorption was induced by irradiation with 355 nm laser pulses. Figure 9a shows transient absorbance spectrum at 1910 cm^−1^ of the four photocatalysts observed in the atmosphere with microsecond time delays. Obviously, the absorbance of the four catalysts at the time origin, 1 µs (time delay ∆t = 1 µs), exhibited different intensities. The initial intensity of the transient absorption originates from photogenerated electrons after excitation and has a rather good relation to the number of the electrons in conduction band and/or shallowly trapped states [35,36]. Moreover, the quantity of the photogenerated charge carriers is dominated by intrinsic, bulk properties of the catalysts. MTiO_2_/Ala-DDA exhibited the strongest initial absorption indicates the existence of a large amount of charge carriers. On the contrary, TiO_2_/Ala appeared the lowest value of initial absorption, perhaps because brookite shows too weak infrared absorption to be detected. Shen et al. confirmed that rutile did not show any transient MIR absorption on the microsecond time scale [37]. Thus, P25 with mixed phases of anatase and rutile also appears a low initial absorption. However, the absorption intensity of MTiO_2_/DDA as pure anatase is also weaker than of MTiO_2_/Ala-DDA, due to formation oxygen vacancies of tetrahedral Ti^4+^ sites created a shallow donor state below the conduction band of MTiO_2_/Ala-DDA [38]. Mid-IR light shows the electrons which are exist in the shallow traps [37]. The photogenerated charge carriers play a key role in the photocatalytic reaction as they have more opportunities to react with the surface adsorbed molecules.

Figure 9b shows the normalized absorbance decay to trace recombination for time delays of 1–400 μs. MTiO_2_/Ala-DDA and P25 exhibited slower decay rate, while the decay rates of MTiO_2_/DDA and MTiO_2_/Ala were faster. It is indicated that the lifetime of the long-lived photogenerated electrons in the microsecond timescale for MTiO_2_/Ala-DDA and P25 are much longer. For P25, the photoelectrons in anatase can transfer rapidly to rutile due to the synergism between anatase and rutile on the surface. Thus, the separation of electrons and holes is effectively realized. However, the shallow donor state produced by oxygen vacancies of tetrahedral Ti^4+^ sites can more effectively promote charge separation besides capturing electrons in MTiO_2_/Ala-DDA. As a consequence, more long-lived photogenerated charges lead to highly efficient photocatalytic activity [39], which is inconsistent with the above photocatalytic performances under UV light.

### 3.4. The Photocatalytic Mechanism

**Scheme 2:**
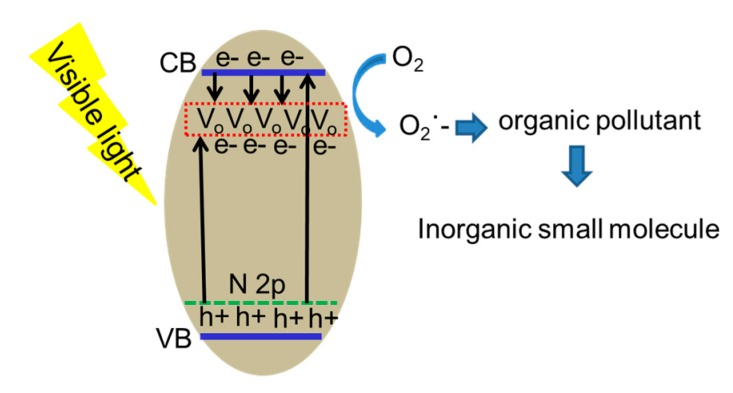
Suggested mechanism for the photocatalysis of MTiO_2_/Ala-DDA under visible light.

The synergistic effect of N-doping and stable oxygen vacancies in MTiO_2_/Ala-DDA may contribute to the improvement of photocatalytic activity under visible light. First, the 2p orbital doped with N increases the valence band (VB) position, while the oxygen vacancy state in MTiO_2_/Ala-DDA lies below the conduction band (CB), which will improve the absorption of visible light. Second, a shallow donor state was created by oxygen vacancies below the conduction band can capture a large number of photogenerated electron, thus increasing opportunities to react with the surface adsorbed molecules. Then, photogenerated electrons oxidized adsorbed oxygen in the surface of MTiO_2_/Ala-DDA to superoxide radicals (O_2_^•^−), and further mineralized organic pollutant to form inorganic small molecules, as shown in Scheme 2.

## 4. Conclusions

In summary, we developed a new and facile chemical process to synthesize N-doped TiO_2_ nanospheres with tetrahedral Ti^4+^ sites in the presence of self-assembling dodecylamine and l-alanine acids. During the synthetic process, dodecylamine—a neutral surfactant—controlled the formation of TiO_2_ spherical structure, while l-alanine acids provided the nitrogen source. The XPS results confirmed the existence of tetrahedral Ti^4+^ sites in N-doped TiO_2_. The sample with tetrahedral Ti^4+^ sites in N-doped TiO_2_ exhibited remarkable photocatalytic performance for degradation of model dye (MB) under whether UV light or visible light. The electron decay results showed that enhanced photocatalytic performance could be attributed to a large amount of photogenerated charge carriers and efficient charge separation. It is demonstrated that the shallow donor state produced by oxygen vacancies of tetrahedral Ti^4+^ sites can effectively promote charge separation and capturing electrons.

## Supplementary Materials

The following are available online at https://www.mdpi.com/2079-4991/9/5/698/s1, Figure S1: Size distributions of MTiO_2_/Ala-DDA, Figure S2: A comparison of photocatalytic degradation of phenol under visible light (The initial concentration of phenol was 5 ppm (50 mL) and the amount of the photocatalyst used was 25 mg).
