# Supplementary material for: Efficient Charge Carrier Separation in l-Alanine Acids Derived N-TiO2 Nanospheres: The Role of Oxygen Vacancies in Tetrahedral Ti4+ Sites
Source: Nanomaterials (Basel). 2019 May 5;9(5):698. doi: 10.3390/nano9050698 (PMC6566907; doi:10.3390/nano9050698)
Supplement: Supplementary file 1 [file nanomaterials-09-00698-s001.pdf]

Supporting Information

Efficient Charge Carrier Separation in L-Alanine Acids Derived N-TiO_2 Nanospheres: The Role of Oxygen Vacancies in Tetrahedral Ti^{4+} Sites

Yongjuan Chen, Xiu Luo, Yao Luo, Peiwen Xu, Jiao He, Liang Jiang, Junjie Li, Zhiying Yan and Jiaqiang Wang *

School of Chemical Sciences & Technology, National Center for International Research on Photoelectric and Energy Materials, Yunnan Provincial Collaborative Innovation Center of Green Chemistry for Lignite Energy, Yunnan Province Engineering Research Center of Photocatalytic Treatment of Industrial Wastewater, The Universities' Center for Photocatalytic Treatment of Pollutants in Yunnan Province, Yunnan University, Kunming 650091, China

* Correspondence: jqwang@ynu.edu.cn; Tel.: +86-871-65031567

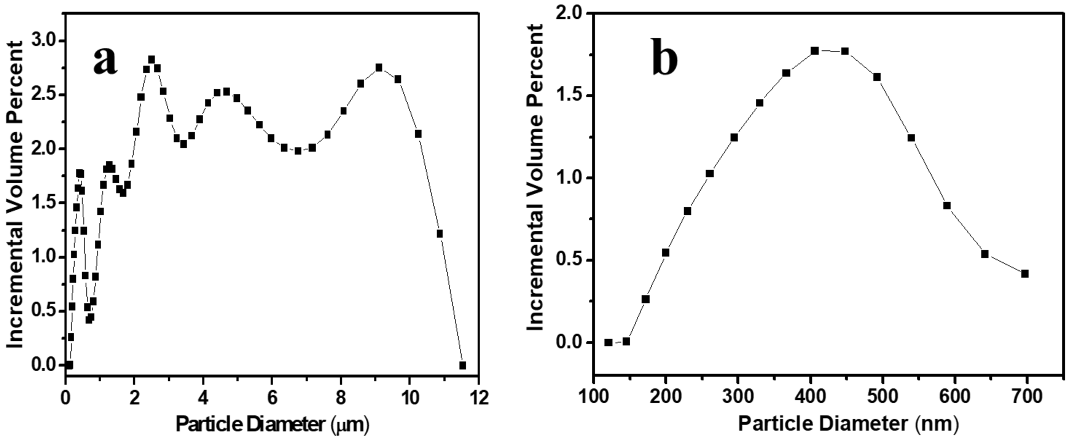

Figure 1. Size distributions of $MTiO_2$/Ala-DDA.

Particle size distribution measurements were conducted at environmental atmosphere with a High Definition Digital Particle Size Analyzer (Saturn DigiSizer II 5205, Micromeritics). Before test, appropriate amount of sample was ultrasonically dispersed in 20 mL deionized water for 20 min, and then a certain amount of dispersion was taken for particle size analyze.

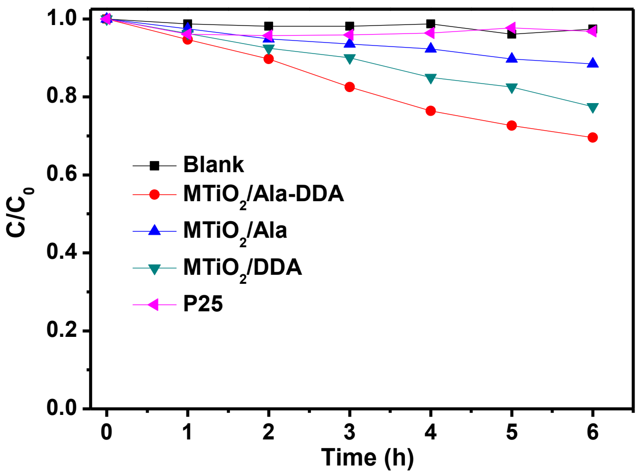

Figure 2. A comparison of photocatalytic degradation of phenol under visible light (The initial concentration of phenol was 5 ppm (50 mL) and the amount of the photocatalyst used was 25 mg).
